# The Effects of Immune System Modulation on Prion Disease Susceptibility and Pathogenesis

**DOI:** 10.3390/ijms21197299

**Published:** 2020-10-02

**Authors:** Neil A. Mabbott, Barry M. Bradford, Reiss Pal, Rachel Young, David S. Donaldson

**Affiliations:** The Roslin Institute & Royal (Dick) School of Veterinary Studies, University of Edinburgh, Easter Bush, Midlothian EH25 9RG, UK; barry.bradford@roslin.ed.ac.uk (B.M.B.); R.Pal@sms.ed.ac.uk (R.P.); rachel.young@roslin.ed.ac.uk (R.Y.); david.donaldson@roslin.ed.ac.uk (D.S.D.)

**Keywords:** prions and prion disease, immune system, inflammation, aging, co-infection, susceptibility

## Abstract

Prion diseases are a unique group of infectious chronic neurodegenerative disorders to which there are no cures. Although prion infections do not stimulate adaptive immune responses in infected individuals, the actions of certain immune cell populations can have a significant impact on disease pathogenesis. After infection, the targeting of peripherally-acquired prions to specific immune cells in the secondary lymphoid organs (SLO), such as the lymph nodes and spleen, is essential for the efficient transmission of disease to the brain. Once the prions reach the brain, interactions with other immune cell populations can provide either host protection or accelerate the neurodegeneration. In this review, we provide a detailed account of how factors such as inflammation, ageing and pathogen co-infection can affect prion disease pathogenesis and susceptibility. For example, we discuss how changes to the abundance, function and activation status of specific immune cell populations can affect the transmission of prion diseases by peripheral routes. We also describe how the effects of systemic inflammation on certain glial cell subsets in the brains of infected individuals can accelerate the neurodegeneration. A detailed understanding of the factors that affect prion disease transmission and pathogenesis is essential for the development of novel intervention strategies.

## 1. Introduction

Prion diseases, also referred to as transmissible spongiform encephalopathies, are subacute, infectious, neurodegenerative diseases that affect humans and some domestic and free-ranging animal species to which there are no effective treatments. A characteristic feature of the prion diseases is the accumulation of PrP^Sc^ (abnormally folded isoforms of the mammalian host’s cellular prion protein, PrP^C^) in affected tissues [1]. The accumulation of PrP^Sc^ in the central nervous system (CNS) ultimately leads to the development of spongiform pathology (vacuolation) and neurodegeneration. A unique feature of these diseases when compared to other neurodegenerative disorders is their transmissibility. Prion infectivity co-purifies with PrP^Sc^ implying that prion particles are mostly, if not entirely, comprised of infectious proteins [2].

The cellular PrP^C^ glycoprotein is encoded by the *PRNP* gene and is expressed on the surface of most cell types via its glycosylphosphatidylinositol anchor. In the CNS, *Prnp* is expressed predominantly in neurons, astrocytes and oligodendrocytes when compared to microglia ([3]; https://www.brainrnaseq.org/), and expression of the PrP**^C^** protein may be important in maintaining myelin homeostasis [4]. Despite this widespread expression, the precise function of PrP^C^ remains the subject of much debate. However, transgenic mice in which the *Prnp* gene is ablated (*Prnp*^−/−^ mice) are fertile and appear to be developmentally normal [5,6]. Likewise, Norwegian dairy goats that lack PrP^C^ expression due to the presence of an early stop-codon mutation in their *PRNP* gene are also healthy [7]. In humans, several naturally-occurring loss-of-function mutations in *PRNP* have been identified and appear to be tolerated [8]. Cellular PrP^C^ is also expressed in most immune cell lineages and may act as an uptake receptor for certain pathogens [9], or modulate cell phenotype [10]. However, although PrP^C^ is expressed highly in key cell populations within the B cell follicles of the secondary lymphoid organs (SLO), PrP^C^ deficiency has little impact on the induction of antigen-specific antibody responses [11].

Expression of PrP^C^ is, however, essential for prion replication in host cells [5]. During prion disease, important post-translational changes occur to the structure of the PrP^C^ molecule that lead to the formation of PrP^Sc^ [12]. These changes affect the physicochemical and biologic characteristics of the PrP molecule, such that prion disease-specific PrP^Sc^ is relatively resistant to proteinase digestion (when compared to PrP^C^), can form insoluble aggregates and can induce the autocatalytic conversion of further copies of PrP^C^ into PrP^Sc^.

To date, a variety of different types of prion diseases have been described. The spontaneous prion diseases, such as sporadic Creutzfeldt–Jakob disease (sCJD) in humans, appear to have an unknown aetiology. This disease typically affects elderly individuals (>60 years old), with a worldwide incidence of approximately 1 case/million population/year. Disease is considered to arise through the spontaneous generation and accumulation of prions in the brains of affected individuals. Some prion diseases can be inherited, and are linked to specific mutations in the *PRNP* gene that appear to predispose the individuals that carry these mutations to prion diseases such as Gerstmann–Sträussler–Scheinker disease (GSS) and fatal familial insomnia [13]. Prion diseases can also be acquired and transmitted between individuals, including natural sheep scrapie, chronic wasting disease in cervid species and bovine spongiform encephalopathy (BSE) in cattle. The demonstration that consumption of BSE-contaminated food during the UK BSE epidemic was responsible for the occurrence of variant Creutzfeldt–Jakob disease (vCJD) in younger people (median age of onset ~29 years old) [14,15] showed how some prion diseases could have zoonotic potential with important consequences for human health. Although sCJD is not considered to be an acquired disease, accidental iatrogenic transmissions have been recorded: for example after the transplantation of tissues (dura mater grafts) or tissue products (pituitary-derived human growth hormones) derived from the brains of sCJD-infected donors, and the use of contaminated surgical instruments [13]. Examples of accidental iatrogenic vCJD transmissions have similarly been reported due to the transfusion of blood or blood products from vCJD-infected donors (for a detailed account of the transmission of prions between species, see [16]).

An understanding of the factors that can affect prion disease transmission and pathogenesis is important for managing disease risk and the development of effective treatments. Early studies in mice revealed that some prion strains accumulated to high levels within days in SLO after peripheral injection, and before the prions had spread to the brain [17]. Subsequent studies showed that specific antibody responses to agents responsible for prion diseases were not induced, despite the high burdens of prions within the SLO in infected mice [18,19,20]. With hindsight, we now know that this lack of an anti-prion (PrP^Sc^-specific) antibody response is most likely due the tolerance of the immune system to cellular PrP^C^. However, although prion infections do not induce strong prion-specific immunity in the majority of infected individuals, the interactions between the prions and certain immune cell populations are essential for disease development, whereas interactions with some immune cells can provide host protection. For example, the study by Eklund and colleagues was the first to suggest that cells within the SLO may actually be sites of prion replication [17]. Soon afterwards, other studies revealed that this peripheral phase of prion replication in SLO such as the spleen was important for the efficient transmission of disease to the CNS [21]. These studies were soon accompanied by others that showed how modulation of the immune system around the time of peripheral exposure could have a profound influence prion disease pathogenesis [22,23,24,25].

Here we discuss how the peripherally-acquired prions exploit certain tissues and immune cells to establish infection. We focus on the peripherally-acquired prion diseases such as natural sheep scrapie, chronic wasting disease in cervid species and BSE in cattle, as these are considered to be transmitted by the oral route through the ingestion of food or pasture contaminated with prions. The consumption of food contaminated with BSE prions was similarly the original cause of vCJD in humans (for an in-depth review on transmission routes, see [16]). We also provide a detailed account of how inflammation, ageing and pathogen co-infection can have a significant impact on prion disease pathogenesis and susceptibility by causing changes to the abundance, function and activation status of specific immune cell populations.

## 2. Splenectomy before Intraperitoneal Prion Infection Extends Survival Times

**Figure 1:**
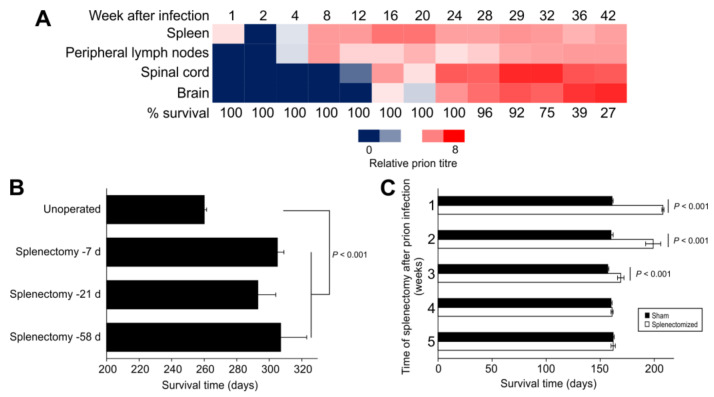
The spleen is an important early site of prion replication and neuroinvasion after peripheral infection. (**A**) In 1967, Eklund et al. [17] showed that high levels of RML scrapie prions accumulated in the spleens of Swiss mice within days after subcutaneous injection, and this occurred before the prions were detected in the spinal cord and brain. Heatmap shows negative log10 of dilution of tissue suspension that contained 1 LD_50_/30 µL when injected intracerebrally into recipient mice. (**B**) In 1970, Fraser and Dickinson [21] showed that splenectomy before IP injection of C57BL/Dk mice with ME7 scrapie prions significantly extended the survival times. Bars, mean ± SEM; *n* = 3–22 mice/group. (**C**) In 1989, Kimberlin and Walker [31] concluded that splenectomy did not extend survival times once infection was established in the spinal cord. In their experiment Compton White mice were IP injected with 139A scrapie prions and the splenectomy performed at the times indicated after prion injection. Bars, mean ± SEM; *n* = 4–9 mice/group.

At the time of writing, five decades have passed since it was originally revealed that the actions of the host immune system could modulate prion disease pathogenesis and susceptibility. The immune system is essential in providing protection against infections with many conventional pathogens. However, quite the opposite has been shown to occur in animals infected with prions by peripheral routes of exposure such as the peritoneal cavity, by subcutaneous injection or via the gastrointestinal tract. Experiments revealed that high levels of scrapie prions accumulated in the spleens of mice within days after peripheral injection and this occurred before the detection of prions in CNS tissues [17,26] (Figure 1A). The impact that this early, non-CNS, prion accumulation had on disease pathogenesis was not known at the time and investigated in a subsequent set of seminal experiments. In that study, the spleens were removed from mice (splenectomy), and once healed, they were subsequently infected with prions [21]. The spleen contains many specialized immune cell populations that are localized in specific niches and these play an important role in providing protection against systemic pathogens, and the removal of their antigens and toxins from the blood-stream [27]. Contrary to these properties, splenectomy prior to, or up to 60 days after an intraperitoneal (IP) prion infection, significantly delayed the onset of the clinical signs of disease [21] (Figure 1B). This revealed that rather than providing host protection, the spleen and other elements of the immune system may conversely play an important role in the establishment of some prion infections. Additional experiments revealed that splenectomy had no effect on disease pathogenesis when the prions were injected with prions directly into the CNS by intracerebral (IC) injection [21]. This finding indicated that the spleen was not simply generating adaptive immune responses that directly caused neuronal damage in the brain. Many follow-up studies have since reinforced this conclusion, and shown that prion disease pathogenesis after IC injection is unaltered in the absence of T cells and B cells [28,29,30].

Analysis of the build up or accumulation of prions in various tissues of mice after peripheral routes of exposure revealed that SLO such as the spleen were amongst the first to be targeted by prions [17]. Soon afterwards, the infection was also detectable in the spinal cord and at later stages in the brain (Figure 1A). Chronological analyses of natural host species, including sheep, cattle and cervids, infected with prions by peripheral routes of exposure have also demonstrated similar disease kinetics [32,33,34,35]. Importantly, these studies raised the suggestion that the initial accumulation of the prions in SLO such as the spleen was required for them to efficiently infect the nervous system, a process that was termed neuroinvasion. Indeed, comparison of the effects of splenectomy and disease kinetics revealed that the pathogenesis became independent of the spleen once infection was established in the spinal cord [31] (Figure 1C). Contemporary studies have since demonstrated the requirement for early prion accumulation in the local draining lymph nodes after prion infection via skin lesions [36], and the Peyer’s patches in the small intestine after oral exposure [37,38,39,40]. This requirement for initial targeting of certain prion strains to the SLO to establish infection has been termed lymphotropism. It is important, however, to mention that examples of non-lymphotrophic prion agent strains exist. Infection with non-lymphotrophic prion strains such as sCJD in humans do not appear to involve significant involvement of the SLO [16]. While it is plausible that sCJD may arise due to the spontaneous misfolding of prions in the brains of affected individuals, accidental iatrogenic transmissions of sCJD (iatrogenic CJD) in humans have occured between individuals via peripheral routes [16]. Since BSE in infection in cattle also appears to have little SLO involvement [16], this suggests that BSE prions have increased tropism for the bovine nervous system. This may potentially negate the need for BSE prions to be processed and amplified within bovine SLO to establish infection of the nervous system.

The findings from the above studies raised the suggestion that treatments that prevented the early build up of certain prion lymphotrophic strains within SLO might help reduce disease transmission, for example by delaying or even preventing the spread of prions to the CNS. Those original experiments reported in the late 1960s and early 1970s, undertaken before prions were proposed and identified, stimulated an exciting and active period of research as tools such as transgenic and “knock-out” mice became available. These studies have identified many of the cellular components that the prions exploit to accumulate in SLO, and shown how modulation of the function or activation status of the cells in these tissues can affect disease pathogenesis and susceptibility. Over the years there have been many useful reviews that have described how prions exploit specific cell populations within the SLO to establish host infection [16,41,42,43,44,45]. Consequently, only the main studies are briefly described in this review, and we refer readers to the above publications if they would like to read further details. We instead focus on how immune stimulation, immunosuppression, pathogen co-infection and changes to the abundance, function or activation status of key immune cell populations can affect prion disease pathogenesis and susceptibility.

## 3. Immune Stimulation Accelerates, Immunosuppression Delays

The suggestion that the activation status of the host immune system could modulate the kinetics of a peripherally-acquired prion disease is not a recent concept and was first recognized in the 1970s. However, once again, the effect of immune stimulation and immunosuppression on prion disease pathogenesis where contrary to the effect such treatments might have been following infection with other pathogenic microorganisms. These studies showed that treatment with immune stimulants such as the mitogen phytohaemagluttinin (PHA) [22] or BCG extract [23] could each accelerate the rate of onset of prion disease after infection by the IP route, and in the case of PHA significantly increase disease susceptibility [22]. In hamsters, the effects of intraperitoneal adenovirus infection on macrophages were similarly associated with a 20% reduction in disease duration when compared with animals infected only with prions [46]. Conversely, treatment with the anti-inflammatory steroid prednisone acetate extended survival times and reduced disease susceptibility [47]. Other anti-inflammatory treatments such as arachis oil or dextran sulphate 500 (single IP injection of 250 µg) similarly delayed disease pathogenesis [24,25]. One study proposed that the reduced susceptibility of mice to IP scrapie prion infection given daily injections with the immune stimulatory CpG oligodeoxynucleotides (for 4 or 20 days after infection) was mediated through such actions on mononuclear phagocytes [48]. However, an independent follow-up study revealed that the repeated CpG treatment used in the above study [48] caused gross disturbances to the microarchitecture of the SLO, including ablation of the FDC networks [49], the key sites of prion replication in these tissues.

At the time that many of the above studies were undertaken, little was understood of the underlying mechanisms responsible for the effects these treatments had on prion disease. However, these treatments were only effective when administered within a short time window immediately before or after the mice were injected with prions by the IP route. This implied that these treatments were modulating the abundance or activity of cells that may aid the propagation of the prions from the peritoneal cavity to their initial replication sites in the spleen. Alternatively, they could also affect the activity of phagocytic cells that could sequester and destroy the prions in the vicinity of the injection site or as they arrive in the spleen (see Section 6.3.).

## 4. Major Histocompatibility Complex (MHC)

The MHC class I and MHC class II molecules encoded by genes in the MHC complex enable short peptides from antigens, either endogenous or pathogen derived, to be displayed on the surfaces of host cells. MHC class I is expressed on all nucleated cells, whereas MHC class II is predominantly expressed on antigen presenting cells. T cells have receptors that specifically recognize these antigen-derived peptides in association with the MHC molecule, the consequence of which is to mount an antigen-specific immune response. Variants in MHC gene allele expression and certain polymorphisms can affect the efficacy of the immune response and are important in the susceptibility to many infectious diseases [50].

Patients with vCJD were reported to have a significantly reduced frequency of the MHC class II type HLA-DQ7 compared to sCJD patients and controls [51]. This raised the suggestion that certain MHC class II molecules may have a direct role in vCJD pathogenesis, or alternatively, that MHC class II type HLA-DQ7 may be more effective at initiating a protective immune response following vCJD infection. However, an independent follow-up study that analysed the same and additional vCJD patients was unable to find any significant association between vCJD patients and MHC type [52]. Genetic deficiency in MHC class I and MHC class II in mice also had no effect on disease duration and susceptibility after IC injection with the Chandler mouse scrapie strain [53]. Together, these data indicate that variants in MHC type do not influence prion disease susceptibility.

## 5. Prions First Replicate upon Follicular Dendritic Cells in SLO

**Figure 2:**
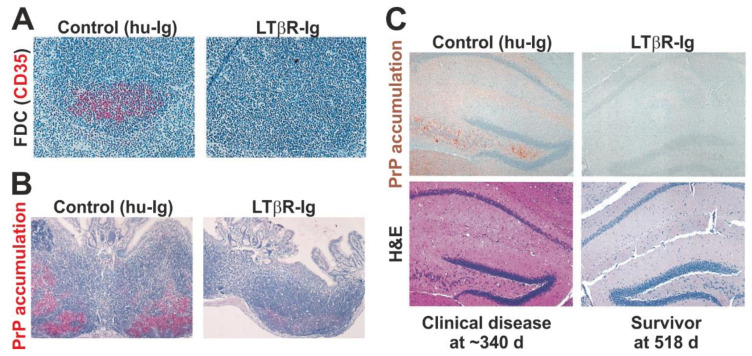
Oral prion disease pathogenesis is impeded in the transient absence of follicular dendritic cells (FDC) at the time of infection [61]. (**A**) Immunohistochemical (IHC) analysis shows that the treatment of mice with a soluble lymphotoxin β receptor (LTβR-Ig) transiently ablates FDC (CD35+ cells, red) in secondary lymphoid tissues. (**B**) Prion accumulation (here shown as disease-specific PrP accumulation by IHC, red) is blocked in Peyer’s patches in the absence of FDC at the time of oral prion infection. (**C**) Oral prion disease susceptibility is blocked in the absence of FDC at the time of oral prion infection. Upper images show IHC detection of disease-specific PrP (brown) and lower H&E-stained panels show presence of prion disease-specific vacuolation (spongiform pathology) in the brains of clinically-affected control mice. All sections counterstained with haematoxylin (blue). Adapted with permission from the American Society for Microbiology from [61] (*J. Virol.* 2003; 77:6845–6854. https://doi.org/10.1128/JVI.77.12.6845-6854.2003).

Mouse studies have shown that within days of peripheral infection, certain lymphotrophic prions accumulate upon the surface of stromal follicular dendritic cells (FDC) within the B cell follicles of SLO [54]. In the absence of FDC, the accumulation of prions in the spleen is blocked and disease susceptibility reduced [29,30,55,56] (Figure 2). These and many other subsequent studies have illustrated how the early targeting of certain prion strains to FDC is essential for efficient establishment of infection, and highlight a critical rate-limiting step in the neuroinvasion process. Indeed, data also suggest that the initial targeting of lymphotrophic prion strains to FDC is important to enable their adaptation to the host environment and to amplify them above the threshold required for neuroinvasion [57,58,59,60].

### 5.1. FDC Trap Prions in a Complement-Dependent Manner

FDC express high levels of complement receptors 1 and 2 (CR1/CD35 and CR2/CD21) and use them to trap and retain intact antigens on their surface within antibody- and/or complement component-opsonized immune complexes. The long-term retention of antigens on FDC enables B cells to generate effective antigen-specific antibody responses [62,63,64]. FDC similarly trap and retain prions on their surfaces as complement-bound complexes [65,66,67,68,69,70,71].

The transient ablation of FDC or depletion of opsonising complement components such as C3 can each impede prion accumulation in the spleen, delay neuroinvasion, and in some instances these defects can reduce disease susceptibility [61,65,72,73,74,75]. This suggests that factors affecting the size and abundance of FDC in SLO such as active immunisation [76] and LPS exposure [77], or their ability to trap and retain immune complexes, could have a significant impact on susceptibility to peripherally-acquired prion infections.

### 5.2. Ageing Affects FDC and their Ability to Trap Prions

As we age, our immune systems become less effective (termed immunosenescence) and the changes this causes correlate with the reduced efficacy of vaccines in elderly individuals, an increased susceptibility to infections, as well as the increased incidence of cancer and autoimmunity. However, just as immunosuppression can impede prion pathogenesis (Section 3), immunosenescence may have a similar effect. Several studies have shown that aged mice (≥18 months old) have reduced susceptibility to peripheral prion infections administered via the IP, intravenous and oral routes [59,78,79]. This reduced susceptibility in the aged mice coincides with disturbances to FDC networks that hinder their ability to trap and retain complement-opsonized immune complexes [78,80,81,82]. The effects of immunosenescence on FDC function may create a significant barrier to susceptibility to peripheral prion infections, especially for cross-species transmissions [59], and may help explain why the majority of clinical vCJD cases in the UK have been identified in young individuals, with relatively few in the elderly [83]. A similar correlation between age, lymphoid follicle size and scrapie susceptibility has also been reported in sheep [84].

### 5.3. PrP^C^ Abundance on FDC Affects Disease Susceptibility

Expression of cellular PrP^C^ is essential for prion replication, and changes to the expression level on host cells can affect disease kinetics [85]. FDC express high levels of PrP^C^ and is essential for the replication of prions on their surface [56,57,86]. As expected, changes to the magnitude of PrP^C^ expression in FDC can affect prion disease pathogenesis. Indeed, when PrP^C^ expression is ablated only in FDC this blocks both prion replication in the spleen and neuroinvasion [57]. Spleens of neonatal mice [87] and aged mice [78] lack PrP^C^-expressing FDC, and this similarly impedes prion replication. Conversely, passive immunisation [88] or active immunisation [76] can each increase FDC abundance, network size and PrP^C^ expression. Furthermore, the increased abundance and size of the FDC in the spleens of mice after active immunisation coincide with increased susceptibility to IP prion infection [76]. The magnitude of the PrP^C^ expressed on FDC may also influence the tropism of prions to SLO [60], and affect the ability of those prions to transmit to other host species [58].

### 5.4. The Distance between FDC and Nerves Is Rate Limiting

The SLO are highly innervated with neurons from both sympathetic and parasympathetic components of the peripheral nervous system. When the prions on the FDC surface are amplified to above the magnitude required to achieve neuroinvasion, they subsequently infect local sympathetic and parasympathetic neurons in the SLO and spread along them to the CNS [54,89,90]. Although the mechanism by which the prions are propagated between FDC and the peripheral nervous system is undetermined, the relative positioning between these cells and the density of sympathetic innervation in the SLO and both directly affect the rate of neuroinvasion [89,91]. This highlights another factor that could affect an individual’s risk of susceptibility to a peripherally-acquired prion infection.

## 6. Propagation of Prions to FDC in Peyer’s Patches

Many natural prion infections are transmitted by the oral route, such as following the ingestion of food or pasture contaminated with prions. After oral exposure, the initial replication of prions upon the FDC in the gut-associated lymphoid tissues (GALT) of the small intestine such as the Peyer’s patches and mature (FDC-containing) isolated lymphoid follicle is essential for efficient neuroinvasion [37,38,39,40]. A series of studies in mice has revealed how the prions exploit specific innate immune cell populations to establish infection upon FDC in the GALT. Furthermore, factors that modify the abundance or function of these cells can significantly alter susceptibility to orally-acquired prion infections.

### 6.1. M Cells are the Gate Keepers of Prions in the Intestine

A single layer of epithelial cells (enterocytes) connected by tight junctions helps to protect the body from the lumenal contents of the intestine. However, the specialized epithelial layer that covers the GALT, the follicle-associated epithelium (FAE), contains a unique population of phagocytic enterocytes known as M cells (reviewed in [92]). Through a process called transcytosis, these cells constitutively sample the lumenal contents of the intestine and transfer particles and pathogens across the FAE to the leukocytes and lymphocytes beneath it in their basolateral pocket structures. The transcytosis of particulate antigens by M cells is an important initial step in the induction of antigen-specific mucosal immune responses to certain pathogens and their toxins, and may also help maintain homeostasis in the commensal gut microbiome [92,93].

**Figure 3:**
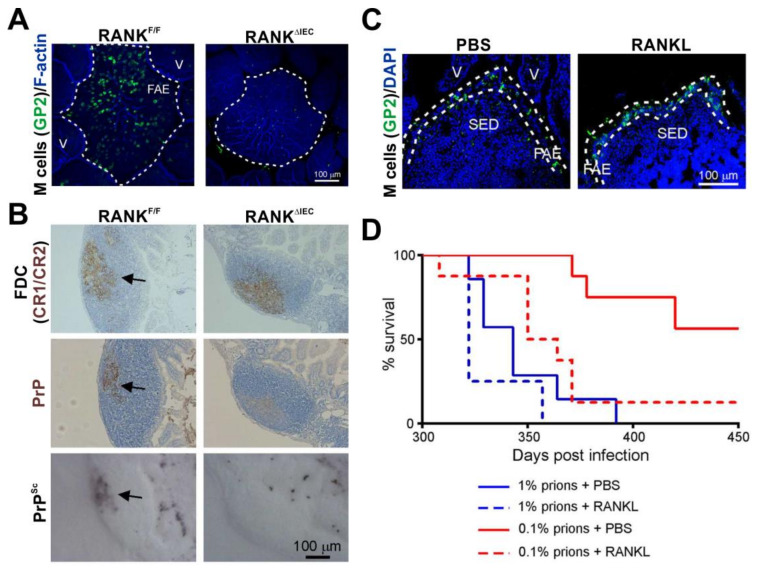
The density of M cells in the epithelia covering the Peyer’s patches directly influences oral prion disease susceptibility [96]. (**A**) Immunofluorescence microscopy shows that RANK^Δ^^IEC^ mice specifically lack M cells (GP2+ cells, green) in the follicle-associated epithelia (FAE) of their Peyer’s patches. F-actin, blue. V, villus. (**B**) In the absence of M cells the accumulation of prions (PrPSc, black and PrP, brown, arrows) upon FDC (CR1/CR2+ cells, brown) in the Peyer’s patches of RANK^Δ^^IEC^ mice is blocked. (**C**) Immunofluorescence microscopy shows that systemic treatment of C57BL/6J mice with the cytokine RANKL increases the abundance of M cells (GP2+ cells, green) in the FAE. DAPI, cell nuclei, blue. SED, subepithelial dome. (**D**) RANKL treatment significantly increases susceptibility to oral prion infection by ~10X. PBS/1% vs. RANKL/1%, *p* = 0.120; PBS/0.1% vs. RANKL/0.1%, *p* < 0.0078; PBS/1% vs. RANKL/0.1%, *p* = 0.205; Log-rank [Mantel–Cox] test). Adapted from [96] under the terms of the Creative Commons Attribution Licence (CC-BY-4.0; https://creativecommons.org/licenses/by/4.0/).

The ability of M cells to transcytose particles from the gut lumen into the GALT has been exploited by some orally-acquired pathogens as an efficient route of infection into host tissues. Prions also appear to exploit these characteristics. Within an hour after oral infection prions can be detected within M cells in the FAE of small intestinal Peyer’s patches, and the absence of these cells at the time of oral exposure blocks disease transmission [54,94,95,96] (Figure 3A,B). Thus, M cells in the FAE overlying the GALT appear to be the initial gate keepers of oral prion infections in the small intestine.

Certain pathogens, such as the bacterium *Salmonella enterica* serovar Typhimurium can specifically manipulate the characteristics of the enterocytes lining the intestine to aid infection, for example by inducing the rapid trans-differentiation of the enterocytes into M cells [97]. Treatments such as bacterial flagellin or exposure to a young commensal microbiota can similarly enhance M cell density in the Peyer’s patches of aged mice [98]. These examples raised the hypothesis that factors that modified the density of M cells in the gut epithelium might also influence oral prion disease pathogenesis. To test this hypothesis, mice were treated with the cytokine RANKL to increase the differentiation and abundance M cells throughout their intestines [99] (Figure 3C). This study showed that the RANKL-mediated increase in the abundance of M cells in small intestinal Peyer’s patches significantly increased susceptibility to oral prion infection by approximately 10 times [96] (Figure 3D).

M cells express high levels of the cellular PrP^C^ on their apical surfaces [100], and this can be used as an uptake receptor for certain pathogenic microorganisms such as *Brucella abortus* [9]. Although pathogen infections and inflammation may enhance PrP^C^ expression in the intestine [101], ablation of PrP^C^ throughout the gut epithelium did not affect oral prion disease pathogenesis [102]. Changes to the expression level of PrP^C^ in M cells are therefore unlikely to affect susceptibility to oral prion infections.

The density of M cells in the Peyer’s patches of aged mice is substantially reduced when compared to young mice [98]. Since oral prion disease is blocked in mice in the absence of M cells [95], the lack of these cells in aged mice most likely contributes to their reduced susceptibility to oral prion infection [78]. However, the age-related decline in M cells could be reversed with young microbiota or administration of certain bacterial ligands [98], suggesting that the reduced susceptibility to oral prion infection in aged animals could be reversed under the right conditions.

Together, these studies show how the modulation of M cell density by factors such as ageing, co-infection with certain gastrointestinal pathogens, inflammation or exposure to microbial stimuli [97,98,103,104,105] may be important risk factors that can increase or reduce oral prion disease susceptibility. For example, if immunosenescence is also associated with a reduced M cell density in elderly humans, this may contribute to their much lower incidence of vCJD cases caused by dietary exposure to BSE prions.

### 6.2. Conventional DC Shuttle Prions to FDC

The mononuclear phagocytes (MNP) arise from hematopoietic precursor cells in the bone marrow and are a heterogeneous population of monocytes, conventional dendritic cells (cDC), and tissue macrophages. The cDC are an entirely distinct lineage from the stromal derived FDC [106] and are strategically located throughout the body to sample the local environment for pathogens and their antigens. After antigen uptake, these cells typically undergo a degree of maturation and migrate toward the draining lymphoid tissue to initiate a specific immune response. Some cDC populations can retain antigens in their native states and transfer them intact to naïve B cells in order to initiate a specific antibody response [107]. The migratory characteristics of cDC are exploited by prions to ensure their efficient transport from the site of exposure to the draining SLO [108,109,110,111]. Mouse cDC and other MNP in the intestine express CD11c (integrin alpha X) highly [112]. In the transient absence of CD11c+ MNP at the time of oral exposure, the early accumulation of prions within Peyer’s patches is blocked and disease susceptibility reduced [108,109]. When the ability of CD11c+ MNP to migrate into B cell follicles was blocked, this similarly impeded oral prion disease [111].

These studies suggest that prions exploit the migratory characteristics of certain CD11c+ MNP populations such as the cDC to ensure their efficient delivery to FDC in Peyer’s patches. Many distinct MNP populations have been identified in Peyer’s patches, and these have been shown to occupy specific anatomical niches [113,114]. The CD8α+ cDC, for example, are localized within the interfollicular regions of the Peyer’s patches [114]. However, factors that specifically affect these cells are unlikely to have significant impact, as the specific deficiency or dysfunction of CD8+CD11c+MNP has no effect on prion disease susceptibility [115,116].

### 6.3. Macrophages Can Destroy Prions

The region of the Peyer’s patches beneath the M cell-containing FAE is known as the subepithelial dome (SED), and contains an abundant and mixed population of cDC and macrophages. Prions can be detected within the macrophages in the SED within a few hours after oral infection [54,94,117]. Tissue macrophages play important roles in host protection against many infectious diseases by phagocytosing and destroying pathogens. In the early 1980s, data from in vitro studies indicated that peritoneal macrophages could similarly phagocytose and degrade scrapie prions, implying a host-protective role in infected animals [118,119]. Consistent with this, the depletion of these cells around the time of oral exposure enhances the accumulation of prions within the Peyer’s patches [120,121].

The ability of tissue macrophages to phagocytose and destroy prions raises the possibility that factors or treatments that stimulate this activity could help reduce oral prion disease transmission. However, macrophages can also provide important homeostatic support to certain cell populations in the intestine such as M cells and enteric nerves [122,123,124]. Impairment of this macrophage-derived support, for example as a consequence of pathogen co-infection or inflammation in the intestine, could indirectly affect M cell function or gut motility and transit time [125,126]. Thus, changes to these homeostatic roles in intestinal macrophages could have a significant impact on the M cell-mediated uptake of prions into Peyer’s patches, or affect their ability to infect enteric nerves.

## 7. Chronic Inflammation Can Facilitate Prion Targeting in Non-SLO Tissues

Chronic inflammation in tissues can induce the formation of granulomas within them. Under some circumstances, these granulomas can contain ectopic germinal centres with networks of PrP^C^-expressing FDC. Studies in mice have shown that the development of granulomas in the kidney, as a consequence of nephritis, can facilitate the replication of prions in these tissues in prion-infected mice and the excretion of infectious prions in urine [127,128]. When induced in the mammary glands of scrapie-affected sheep, for example in response to mastitis, these can similarly act as novel sites of prion replication and transmit prions to suckling lambs via milk [129,130,131]. Lymphotrophic prions can accumulate in subcutaneous soft tissue granulomas in association with an inflammation-dependent PrP^C^-expressing stromal cell population [132], although a subsequent study has suggested a role for a FDC-like cell population [133]. However, not all forms of tissue granulomas appear to be capable of supporting prion accumulation in infected individuals. Granulomas can form in the intestine, for example in the submucosa around the invading larvae of helminth pathogens such as *Heligmosomoides polygyrus*. These granulomas contain abundant populations of MNP, but due to the absence of FDC or other PrP^C^-expressing stromal cells they do not contain PrP^Sc^ deposits in mice orally co-infected with prions [134].

## 8. Pathogen Co-Infections Can Affect Oral Prion Disease

### 8.1. Gastrointestinal Helminths

**Figure 4:**
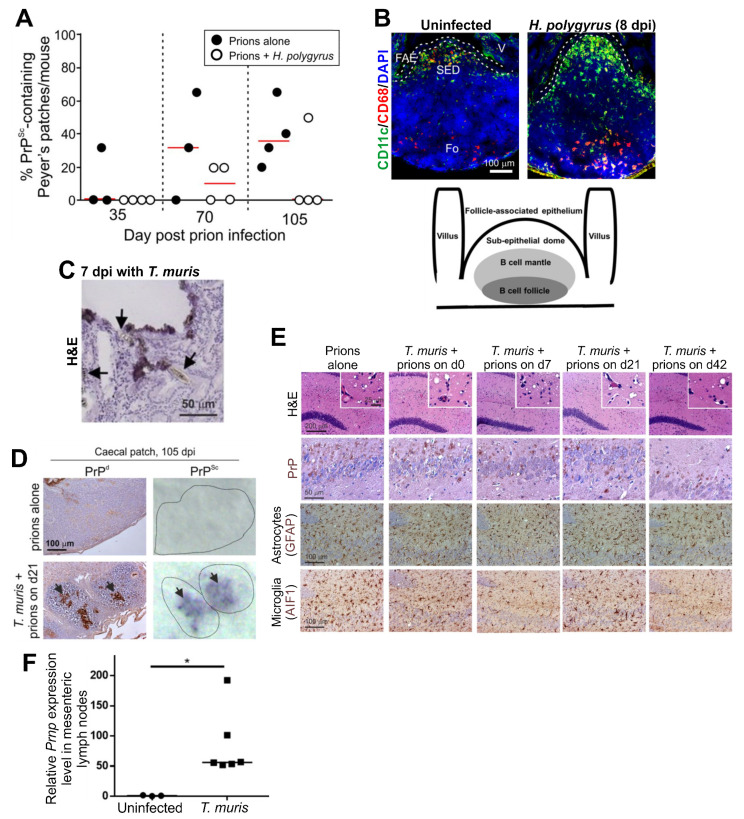
Effect of co-infections with gastrointestinal helminths on oral prion disease pathogenesis in.mice. Panels A and B illustrate the effects of co-infection with *Heligmosomoides polygyrus* in the small intestine [134]. C57BL/6J mice were first orally-infected with *H. polygyrus*. L3 larvae and orally infected with ME7 scrapie prions 8 days later. (**A**) The frequency of PrP^Sc^-containing Peyer’s patches was lower in mice co-infected with *H. polygyrus*. (**B**) Immunofluorescent analysis showed that the distribution of CD11c+ mononuclear phagocytes (green) was disturbed in the Peyer’s patches on d 8 after *H. polygyrus* infection. FAE, follicle-associated epithelium; SED, subepithelial dome; Fo, follicle; V, villus; dpi, days post-infection. Cartoon shows the anatomical boundaries in the Peyer’s patch. Panels C-E illustrate the effects of co-infection with *Trichuris muris* in the large intestine [134]. C57BL/6J mice were first orally-infected *T. muris* infective eggs and orally infected with ME7 scrapie prions and the days indicated after the helminth infection. (**C**) Arrows show the close association of *T. muris* with the caecal epithelium and lamina propria and the damage caused to the epithelium. (**D**) In mice infected with prions alone PrPSc was undetectable in the caecal patches at 105 dpi. However, high levels of PrP^Sc^ were detected in the caecal patches of the mice co-infected with *T. muris*. (**E**) High levels of prion-specific vacuolation (H&E), PrP accumulation, reactive astrocytes (GFAP+ cells, brown) and active microglia (AIF1+ cells, brown) were detected in the brains of mice from each infection group. Counterstain, haematoxylin, blue. (**F**) Upregulated *Prnp* expression in the mesenteric lymph nodes of *T. muris*-infected mice. *, *p* < 0.05. Panels A and B are adapted from [134] under the terms of the Creative Commons Attribution Licence (CC-BY-4.0; https://creativecommons.org/licenses/by/4.0/). Panels C–E are adapted from [40] under the terms of the Creative Commons Unported Licence (CC-BY-3.0; https://creativecommons.org/licenses/by/3.0/).

Mammals are often exposed to and infected with gastrointestinal helminth pathogens in their natural environments. *Trichuris muris* and *H. polygyrus* are natural helminth pathogens of mice, and have been used to study the effects of gastrointestinal helminth infections on oral prion disease pathogenesis. These helminth pathogens affect different niches in the intestine. *H. polygyrus* infection, for example, is restricted to the duodenum of the small intestine and causes pathology to the mucosa within it. Co-infection of mice with *H. polygyrus* around the time of oral prion exposure reduced the early accumulation of prions upon FDC in the Peyer’s patches and extended disease duration [134,135] (Figure 4A). The delayed prion disease pathogenesis in the mice co-infected with *H. polygyrus* was associated with an altered distribution of CD11c+ MNP in their Peyer’s patches with a specific increase in abundance in the B cell mantle region (Figure 4B). This implied that the altered distribution of the cDC in the Peyer’s patches of the co-infected mice may have impeded the ability of these cells to efficiently propagate the prions to the FDC within the B cell follicles [134].

Infection with *T. muris*, in contrast, is restricted to the large intestine, where it causes significant pathology to the epithelium and lamina propria in the caecum (Figure 4C). Co-infection with *T. muris* around the time of oral prion exposure coincided with the earlier accumulation of prions (PrP^Sc^) within the caecal patches (Figure 4D). Despite this effect, all the co-infected mice developed clinical prion disease with similar neuropathology (Figure 4E), survival times and disease susceptibility to mice infected with prions alone [136]. These data are consistent with findings from experimental mice and natural host species such as sheep, goats and white-tailed deer that show that the GALT in the large intestine (such as the caecal patches) are not early sites of prion replication and neuroinvasion after oral infection [32,136,137,138]. The low density of M cells in the FAE overlying the large intestinal GALT [139], in addition to the thick mucus layer covering the epithelium at this site [140], is most likely responsible for the relative inability of the large intestinal GALT to acquire particulate antigens such as prions [136].

### 8.2. Pathogenic Bacteria

Oral infection of mice with the pathogenic bacterium *S.* Typhimurium can cause significant pathology and inflammation in the large intestine. The reduced survival times observed in mice co-infected with *S.* Typhimurium were reported to be a consequence of the effects of colitis on the uptake of prions from the gut lumen, or the upregulated expression of PrP^C^ in the intestine and mesenteric lymph nodes [101]. However, *Prnp* expression is also upregulated in the mesenteric lymph nodes during infection of the large intestine with *T. muris* (Figure 4F) but this was not associated with shortened survival times when co-infected with prions [136]. As mentioned above (Section 6.1), *S.* Typhimurium can inject factors into enterocytes that enhance the abundance of M cells in the gut epithelium to aid infection [97]. Exposure to bacterial flagellin can also increase the abundance of M cells in the FAE [98]. Although not tested in the above study [101], the reduced survival times in the mice co-infected with *S*. Typhimurium may also be a consequence of the effects on the abundance of M cells in the small intestinal Peyer’s patches.

A fascinating study has described how interactions between M cells, a specific population of enteric nerves and components of the commensal gut microbiota can together provide protection against oral *Salmonella* infection [141]. Gut-innervating nociceptors are a specialized subset of sensory neurons that respond to pain and harmful stimuli, and these were found to be closely associated with M cells in Peyer’s patches [142]. Furthermore, stimulation from nociceptors, for example following *Salmonella* infection, could suppress the abundance of M cells in Peyer’s patches and this was accompanied by an increased abundance of the commensal, segmented filamentous bacteria (SFB), on the lumenal surface of the FAE [141]. The combined effects of the nociceptor-mediated reduction in M cell density and increased abundance of SFB on the FAE reduced susceptibility of the mice to oral *Salmonella* infection [141]. This study raises the intriguing hypothesis that nociceptor-mediated stimulation in response to certain pathogenic microorganisms, harmful stimuli, noxious substances or inflammatory mediators in the small intestine could similarly reduce susceptibility to orally-acquired prion infections.

## 9. CNS Prion Disease

Once the prions enter the brain, their build up ultimately leads to the development of the characteristic spongiform pathology and extensive neurodegeneration in targeted brain regions [143]. CNS prion infections are also accompanied by extensive microglial and astrocyte activation in affected regions [144]. The precise mechanism that causes the neurodegeneration during CNS prion disease remains to be fully understood, but as discussed below, CNS inflammation, or the actions of certain immune cell populations or inflammatory mediators can have a significant impact. For example, although the underlying mechanism was not addressed, prion disease was accelerated in infected mice with concurrent inflammation directly within the CNS caused by the induction of experimental allergic encephalitis (EAE, a commonly used mouse model of multiple sclerosis in humans) [145].

### 9.1. The Yin and Yang of the Microglia

The microglia are the resident macrophages of the CNS, and a change in their status from resting to activated is one of the earliest neuropathological features in the brain during prion disease, and occurs before the development of the neuropathology [146,147,148]. The microglia are established by embryonic day 8 in the brain from yolk sac-derived progenitors, and are mostly maintained by self-renewal in a CSF1R-dependent manner [149,150]. Deficiency in the monocyte chemokine receptor CCR2 does not affect microglia abundance or CNS prion disease [151], indicating that the microglial expansion that occurs during prion disease is a consequence of the local proliferation of CNS-resident cells [152]. As the studies described below show, changes to the abundance and phenotype of the microglia in the prion disease-affected brain can affect the rate of the neurodegeneration.

#### 9.1.1. Microglia Can Phagocytose and Destroy Prions in the Steady State

The microglia, just like the macrophages in other tissues, are highly phagocytic cells that help remove apoptotic cells (by a process known as efferocytosis) and provide a first line of defence against pathogens. Intracellular accumulations of prions can be detected within microglia in affected brain regions [153], but these cells are not important sites of prion replication [154]. This apparent inability of the microglia to replicate prions may be a consequence of their reduced expression level of *Prnp*, when compared to neurons and astrocytes [3]. Although it is important to mention that factors other than the magnitude of *Prnp* are likely to play a role, as the transgenic expression of high levels of *Prnp* in T cells and B cells was insufficient to sustain prion replication within them [155,156]. Instead, data suggest that microglia, like the tissue macrophages, similarly provide host protection by phagocytosing and destroying the prions.

**Figure 5:**
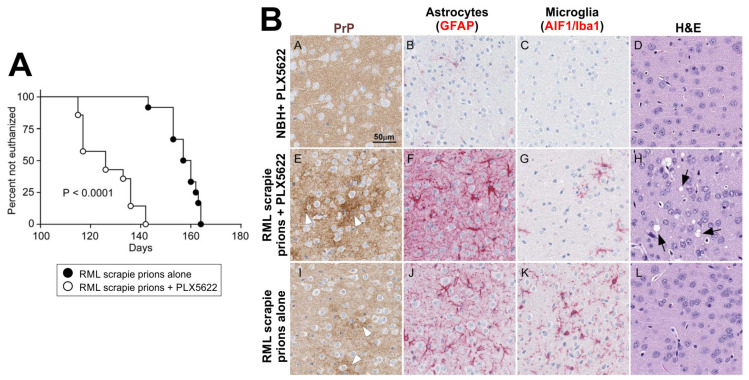
Accelerated CNS prion disease in the absence of microglia. (**A**) Survival curve shows enhanced onset of clinical prion disease in mice treated with the CSF1R-targeting kinase inhibitor PLX5622. (**B**) Immunohistochemical analysis shows increased PrP accumulation (brown), reduced abundance of microglia (AIF1/Iba1+ cells, red), increased reactive astrocyte activation (GFAP+ cells, red) and prion-specific vacuolation (arrows, H&E panel) in PLX5622-treated mice at 100 days after IC injection with RML prions. Adapted with permission from the American Society for Microbiology from [160] (*J. Virol.* 2018;92:e00549-18, https://doi.org/10.1128/JVI.00549-18).

The differentiation, proliferation and survival of microglia and tissue macrophages is controlled by signalling through the cytokine colony-stimulating factor 1 receptor (CSF1R) [157]. Deficiencies in, or pharmacological blockade of CSF1R-signalling, can both block microglia differentiation and survival in the brain. The cytokine IL-34 can also bind to CSF1R, in addition to colony-stimulating factor 1 (CSF1), and stimulation via this cytokine similarly plays an essential role in microglia differentiation [158]. A series of studies have shown that the partial ablation of microglia in ganciclovir-treated CD11b-HSVTK mice (transgenic mice expressing thymidine kinase of Herpes simplex virus via the CD11b (*Itgam*) promoter; [159]), their partial deficiency in IL-34^-/-^ mice [159] or their partial deficiency after treatment with a kinase inhibitor (PLX5622) that targets CSF1R [160] (Figure 5) can each accelerate CNS prion disease. These effects coincided with the increased accumulation and deposition of prions in the brain, indicating that the microglia are host protective during CNS prion disease by phagocytosing and destroying prions. However, some important caveats should be considered. Peripheral macrophage and mononuclear phagocytes populations are also ablated to differing extents in the above ablation models, and the influence this has on disease pathogenesis should be considered. For example, although PLX5622 has been widely used to specifically ablate microglia, this treatment also causes long-term effects on the turnover and function of bone marrow-derived, circulating, and tissue-resident macrophages [161]. High levels of lymphotrophic prions also accumulate in the SLO even after their direct injection into the brain by IC injection [56] and subsequently spread back to the brain [59,80]. As the accumulation of prions is enhanced in SLO in the absence of tissue macrophages [120,121], their absence in the above models may have contributed to the increased accumulation of prions in the CNS. It is also plausible that the ganciclovir-mediated ablation of the microglia in transgenic CD11b-HSVTK mice triggered an inflammatory cytokine response in the brain, and this may have stimulated a neurotoxic profile in the remaining microglia or other glial cells.

#### 9.1.2. Microglia Engulf Apoptotic Bodies

Milk fat globule epidermal growth factor 8 (MFGE8) is a secreted factor that binds to exposed phosphatidyl serine residues exposed on the surfaces of apoptotic bodies to facilitate their clearance by phagocytic cells, including microglia. CNS prion disease is accelerated in mice deficient in MFGE8 and coincides with the increased accumulation of prions in the brain [162]. This suggests that the clearance of prions by microglia may be mediated via their indirect removal of infected apoptotic neurons. Phagocytes can bind MFGE8-opsonised apoptotic bodies through interactions with the integrins αvβ3 and αvβ5. Whether these integrins and other related receptors facilitate the phagocytosis of prions by microglia is uncertain. Triggering receptor expressed in myeloid cells-2 (TREM2) also contributes to the phagocytosis of apoptotic neurons and is upregulated in microglia during prion disease [163]. Signal regulatory protein α (SIRPα), conversely, acts as a negative regulator of phagocytosis in microglia and other MNP populations [164]. However, prion uptake by microglia occurs independently of TREM2 and SIRPα, as CNS disease is unaltered in transgenic mice that lack these receptors [163,165]. Sialoadhesin (CD169) specifically binds to sialylated glycoproteins and is expressed by various MNP populations [166], including activated microglia [167]. Although PrP^Sc^ is extensively sialylated [168], deficiency in sialoadhesin similarly does not affect the development of neuropathology during CNS prion disease [169]. Thus, the engulfment of prions by microglia is indirectly mediated via binding to MFGE8 but occurs independently of SIPRα, TREM2 and sialoadhesin.

#### 9.1.3. Microglia Can Cause Neurodegeneration

Despite the proposed host-protective role for the microglia during CNS prion disease, alterations to their activation status can lead to neurotoxicity. Prion infection in the steady state does not evoke a typical pro-inflammatory cytokine response [148,168,170]. The microglial characteristics during the early stages of CNS prion disease are instead similar to the anti-inflammatory profile exhibited by macrophages following their engulfment of apoptotic cells [171]. This is consistent with data showing that CNS prion disease is unaltered in the steady state in mice lacking the NLRP3 inflammasome [172] (essential for release of IL-1β), NF-κB signalling [173] or MyD88 signalling [174]. Treatment of scrapie-affected sheep with the glucocorticoid dexamethasone during the clinical phase similarly had no effect on the development of neuropathology [175]. Modest levels of the anti-inflammatory cytokine TGF-β are expressed in the CNS during prion disease [176,177]. Whether this cytokine is expressed in the prion disease-affected regions of the brain at sufficient levels to constrain the local induction of pro-inflammatory microglial responses, or mediate neurogenic properties [178] remains to be determined. However, it is interesting to note that when the availability of TGF-β in the brain is blocked, this exacerbates the neuropathology [179].

When the accumulation of PrP^Sc^, neurodegeneration and microglial phenotype was compared across brain regions in mouse prion disease models, it was noted that the neurodegeneration was restricted to areas of the brain where PrP^Sc^ accumulation was also associated with an upregulated innate immune response in the microglia [147,148,168]. The precise trigger that mediates the switch in the activation status of the microglia in the steady state is uncertain. However, it is evident that microglia respond to changes in the composition of the carbohydrates displayed on the surface of PrP^Sc^ in such way, that a reduction in sialic acid residue content can induce a pro-inflammatory response [180].

Infection of the brain with prions has been suggested to induce a “primed state” in the microglia that enables these cells to rapidly respond to subsequent exposure to pro-inflammatory stimuli [181]. When the microglia are stimulated in this manner it can have a significant impact on CNS prion disease pathogenesis. For example, central and systemic treatment of mice with LPS during the CNS phase of prion infection triggers the rapid release of pro-inflammatory cytokines and mediators, and this is accompanied by increased microglial activation, increased neurodegeneration and accelerated disease progression [182,183,184]. This suggests that the microglial response during prion disease favours a homeostatic and potentially pro-resolving/anti-inflammatory phenotype. However, when exposed to pro-inflammatory stimuli such as bacterial LPS, this phenotype appears to be lost, polarising the microglia towards an activated phenotype which can heighten the rate of neurodegeneration. Indeed, as the prion infection proceeds in the brain, the homeostatic transcriptomic signatures in the microglia are replaced by neuroinflammatory signatures [168]. These data show how the effects of systemic and central inflammation on microglial status can exacerbate CNS prion disease.

#### 9.1.4. Microglia as Therapeutic Targets

Taken together, these studies raise the suggestion of whether manipulating the abundance or phenotype of microglia may have therapeutic potential during CNS prion disease. One example may include the blocking of environmental cues which stimulate the microglial pro-inflammatory and neurotoxic phenotypes. Ultimately, manipulating the phenotype of the microglia to optimize prion clearance, and likewise the production of pro-resolving factors (including TGF-β) may serve as beneficial factors.

Experiments in mice suggest that such approaches may be beneficial in individuals with a prion infection in the brain, but the wider impacts of long-term administration of immunosuppressive drugs should not be overlooked. One study, for example, has shown how oral treatment with the immunosuppressant FK506 from the onset of the clinical signs was effective in suppressing microglial responses and reducing disease susceptibility in a mouse sCJD model [185]. Inhibition of microglial proliferation or “priming” may also have efficacy. Whereas treatment with the CSF1R kinase inhibitor PLX5622 partially ablates the microglia [160], treatment with the CSF1R-specific kinase inhibitor GW2580 reduces microglial proliferation and skews the cells towards a neuroprotective phenotype [150]. Coincident with the induction of these potentially neuroprotective effects, treatment of mice with GW2580 or an anti-CSF1R blocking antibody, has been shown to slow the rate of neurodegeneration and extend survival times in mice with CNS prion disease [152].

#### 9.1.5. The Commensal Gut Microbiome Constitutively Modulates Microglia Status

The mammalian gastrointestinal tract is colonized by a huge and diverse community of commensal microorganisms and is estimated to include over 1000 different bacterial species. These microorganisms and the metabolites they produce provide a range of beneficial and protective effects on their mammalian host. These include the production of essential nutrients such as vitamins [186], outcompeting with pathogens [187] and aiding the regulation and development of the immune system [98,141,188,189]. An exciting set of studies has also shown how the components of gut commensal microbiota and their metabolites can also modulate the development and function of cells in the CNS, for example by directly stimulating enteric nerves, promoting the release of neuropeptides from enteroendocrine cells or producing important brain bioactive mediators and neurotransmitters including dopamine, serotonin and γ-aminobutyric acid (reviewed in [190,191]).

The commensal microbiota also constitutively regulates the development and function of the microglia in the brain. Germ-free mice that completely lack a commensal microbiota have defects in microglia development, differentiation and function, and their response to LPS stimulation or LCMV infection are diminished [192]. Similar effects on microglia status were also observed when conventionally-housed SPF mice were treated with broad-spectrum antibiotics. Disturbances to the commensal microbiota in the intestine have been proposed as risk factors that affect susceptibility and the pathogenesis of many neurodegenerative disorders including Parkinson’s disease, Alzheimer’s disease, amyotrophic lateral sclerosis and Huntington’s disease (reviewed in [193]). The effects of signals from certain bacteria on T cells in the small intestine can also exacerbate the pathogenesis of EAE in the spinal cords of mice [194], implying a similar pathogenic role for changes to components of the small intestinal microbiota in multiple sclerosis patients.

However, despite the prominent activation and involvement of the microglia in the brain during prion disease, the absence of the commensal microbiota does not affect the development of the neurodegeneration in the steady state [195,196]. Whether the microglia in the brains of prion-infected germ-free mice are less sensitive to subsequent neurotoxic activation by pro-inflammatory stimuli remains to be determined. Treatment of prion-infected mice with the tetracycline antibiotics (doxycycline, minocycline and tetracycline) around the onset of the clinical phase has been shown to extend survival times [197]. Tetracycline antibiotics can suppress cytokine synthesis [198] and this might have a beneficial effect on CNS prion disease by reducing systemic inflammation. However, these antibiotics have also been shown to block PrP^C^–PrP^Sc^ conversion and neurotoxicity [199].

### 9.2. Reactive Astrocytes: Neuroprotective or Neurotoxic?

Astrocytes are important glial cells that provide homeostatic support to neurons in the steady state. For example, these cells can induce the formation of excitatory synapses through production of mediators such as thrombospondins, SPARC-like 1 and glypicans [200,201]. Astrocytes can also prune synapses to refine neural circuitry to ultimately modulate synaptic plasticity [202]. However, dysfunctional and reactive astrocytes can gain neurotoxic properties following brain injury in the ageing brain and in certain neurodegenerative disorders [203,204].

The contrasting neuroprotective and neurotoxic properties of reactive astrocytes imply that, just like the microglia, these cells may similarly have contrasting roles during CNS prion infections: on the one hand providing homeostatic support to infected/damaged neurons, while on the other hand causing neurotoxicity and driving neuronal death. For example, the production of MFGE8 by astrocytes aids the phagocytosis and clearance of prion-infected and apoptotic neurons by microglia in some mouse strains [162]. However, it is important to note that reactive astrocytes are also capable of phagocytosing apoptotic neurons [205], challenging the conclusion that the phagocytosis and clearance of prions in the brain is restricted to the microglia [159,160]. Unlike the microglia [154], astrocytes may also be important sources of prion replication in the brain [206].

The accumulation of misfolded PrP^Sc^ in the brain during prion disease triggers the unfolded protein response (UPR) [207]. This alters the astrocyte secretome, reduces their synaptogenic properties and stimulates the production of neurotoxic factors which together enhance the rate of neurodegeneration [203]. Specifically, phosphorylation of PERK signalling in astrocytes causes the transient shutdown of protein synthesis via phosphorylation of eIF2α. The pharmacological targeting of certain signalling pathways in astrocytes has been shown to have efficacy in reducing hypoxia-induced CNS edema [208]. Similar pharmacological targeting of PERK signalling in astrocytes in mice can provide neuroprotection during CNS prion disease, providing an indication that treatments that modulate the phenotype of the reactive astrocytes may have therapeutic potential [203].

#### 9.2.1. Microglia Can Modify the Phenotype of Reactive Astrocytes

**Figure 6:**
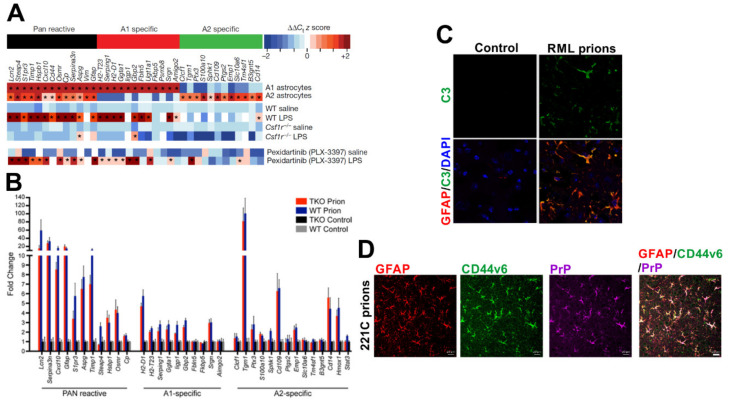
Induction of a mixed A1 neurotoxic and A2 neuroprotective reactive astrocyte phenotype during CNS prion disease. (**A**) Systemic LPS treatment induces an A1 neurotoxic transcriptional signature in reactive astrocytes in the brain. In microglia-deficient *Csf1r*^−/−^ mice the LPS-mediated induction of A1 neurotoxic reactive astrocytes is blocked. However, A1 neurotoxic reactive astrocytes are still induced after microglia-depletion by PLX-3397 treatment (~95% reduction) [211]. (**B**) A mixed A1 neurotoxic and A2 neuroprotective reactive astrocyte transcriptional signature was observed in the reactive astrocytes in the brains of wild-type (WT) mice terminally affected with RML prions. Deficiency in the microglial-derived factors TNF-α, IL-1α and C1qa (TKO) had little influence on the induction of this response [214]. (**C**) Immunofluorescence microscopy shows that GFAP+ (red) reactive astrocytes express high levels of complement component C3 (green) in the prion disease-affected brain [214]. Nuclei detected with DAPI (blue). (**D**) Immunofluorescence microscopy shows that GFAP+ (red) reactive astrocytes with abundant disease-specific PrP (magenta) express high levels of CD44 variant 6 (green) in the prion disease-affected brain [215]. Panel A is adapted from [211] with permission from the Nature Customer Service Centre GmbH (Liddelow SA et al. Neurotoxic astrocytes are induced by activated microglia. *Nature* 2017;541:481-487, https://doi.org/10.1038/nature21029). Panels B and C are adapted from [214] under the terms of the Creative Commons Licences (CC-BY-4.0; https://creativecommons.org/licenses/by/4.0/, and CC0-1.0; https://creativecommons.org/publicdomain/zero/1.0/). Panel D is adapted from [215] under the terms of the Creative Commons Attribution Licence (CC-BY-4.0; https://creativecommons.org/licenses/by/4.0/).

Astrocytes and the microglia can interact with each other in the brain through direct contact and production of secretory factors [209]. For example, astrocyte-derived CSF1/IL-34, TGF-β2 and cholesterol are essential for microglial survival [210]. The reactive astrocytes have been classified into two main functional and transcriptional subclasses. The A1 subclass of reactive astrocytes appear to exhibit neurotoxic properties, whereas the A2 astrocytes produce neurotrophic factors and can provide neuroprotection [211]. A seminal study has shown how contact with microglia and microglia-derived factors is essential for the induction of A1 neurotoxic reactive astrocyte activation [211]. For example, the production of the cytokines TNF-α, IL-1α and complement component C1q by the microglia in response to systemic LPS treatment predominantly induce an A1 phenotype and transcriptomic response in the reactive astrocytes [211]. In the absence of microglia (*Csf1r* ^−/−^ mice) or these microglial-derived factors, the LPS-mediated induction of A1 astrocytes is blocked. The A1 reactive astrocytes that are induced by systemic LPS treatment also have a distinct transcriptomic signature from the A2 subset [211] (Figure 6A). While the administration of LPS does not itself appear to induce significant neurotoxicity, data indicate that neuronal injury is required for these cells to become susceptible to astrocyte-mediated cytotoxicity [212]. Whereas the expression of certain A1 reactive astrocyte-associated genes has been reported during the preclinical phase of prion disease, this polarity is lost as the infection proceeds towards the terminal stage [177,213]. Although the reactive astrocytes in the prion disease-affected brain have been suggested to be neurotoxic [203], they display a mixed A1 and A2 transcriptomic signature [177,214] and notably express high levels of CD44 [215] and complement component C3 [203,214] (Figure 6B–D). Whereas CNS prion disease was unaltered in mice deficient in TNF-α [216,217] or C1q [65,66], the combined deficiency in TNF-α, IL-1α and C1q was shown to accelerate the disease [214]. Although complement C3 expression was reduced in the astrocytes in the infected mice with a combined deficiency in these microglial-derived factors, this had little effect on GFAP expression or the transcriptomic signature of the reactive astrocytes [214] (Figure 6B). This suggests that in the steady state, factors independent of microglial-derived TNF-α, IL-1α and C1q are required for the activation of astrocytes in the prion disease-affected brain.

Further studies are required to determine whether microglia are required for induction of complement component C3+ reactive astrocytes during prion disease. When microglia were partially ablated using the CSF1R-targeting kinase inhibitor PLX5622, the overall expression of A1 and A2 reactive astrocyte-associated transcripts in the brains of mice infected with prions was enhanced [177]. This suggests a potential additional neuroprotective role for the microglia in the prion disease-affected brain by limiting neurotoxic reactive astrocyte activation. However, it is important to note that the retention of just 5% microglia after treatment with the CSF1R-targeting kinase inhibitor PLX3397 was sufficient to induce A1 reactive astrocyte activation after systemic LPS treatment [211] (Figure 6A). Transmissions to mice with a complete and specific deficiency in microglia will help to resolve this issue [218].

#### 9.2.2. Systemic Inflammation and Reactive Astrocyte Activation During Prion Disease

The demonstration that the effects of systemic LPS treatment on microglia can indirectly induce neurotoxic A1 reactive astrocyte activation [211] raises the possibility that pro-inflammatory mediators could have a significant impact on reactive astrocyte phenotype and neurodegeneration during prion disease. Systemic LPS treatment, for example, enhances neuronal apoptosis in the brains of mice infected with prions, and accelerates disease progression [182,183,184]. The effects of systemic LPS treatment on CNS prion disease coincide with the enhanced abundance and inflammatory activation of the microglia, as well as elevated production of the pro-inflammatory cytokines TNF-α, IL-1β and IL-6, and cytotoxic mediators such as nitric oxide [182,183,184]. These experiments were published before the demonstration that LPS treatment also induced neurotoxic A1 reactive astrocyte activation [211]. However, a subsequent study showed that the reactive astrocytes in the brains of infected mice centrally-treated with either TNF-α or IL-1β also had enhanced chemokine responses and increased nuclear localisation of the transcription factor NF-κB [219].

**Figure 7:**
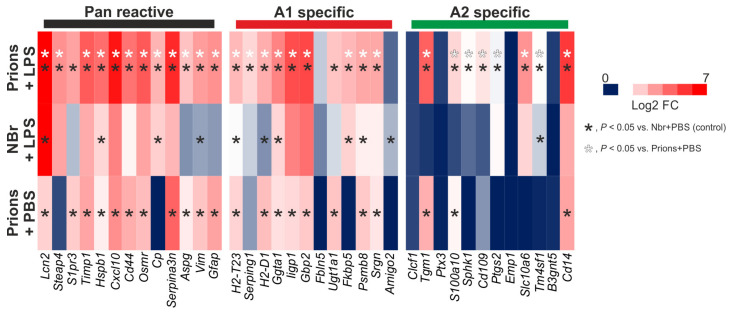
Systemic LPS treatment elevates the expression of pan-, A1 neurotoxic- and A2 neuroprotective reactive astrocyte-associated genes in the hippocampus of mice infected with ME7 scrapie prions. Analysis of a published microarray dataset (NCBI GEO accession no: GSE23182; [184]) of the effects of LPS treatment on astrocyte-associated gene expression in the brains of mice infected with prions. Mice were systemically injected (IP) with at 18 weeks post-IC injection with prions. Hippocampi were removed 6 h later and gene expression compared by microarray. Parallel sets of mice were injected with PBS or normal brain homogenate (NBr) as controls. Heatmap shows log2 fold change when compared to uninfected control mice injected with PBS. *n* = 3 mice/group.

The effects of systemic inflammation on the reactive astrocytes could each have a significant impact on CNS prion disease. To investigate this further we analysed mRNA micro-array expression data from a published study of the effects of systemic LPS treatment on the hippocampus of mice infected with ME7 scrapie prions (NCBI GEO accession no: GSE23182; [184]). Here, the mice were systemically injected (IP) with LPS before the terminal phase of prion disease (at 18 weeks post-IC injection with prions). Parallel sets of mice were injected with PBS or normal brain homogenate as controls. This analysis also revealed a predominantly A1-reactive astrocyte-associated transcriptional signature in the hippocampus during the pre-terminal phase of prion disease (prions + PBS; Figure 7), consistent with published studies [177,213]. Importantly, this transcriptional signature was significantly enhanced after LPS treatment (prions + LPS; Figure 7). As anticipated [211], the systemic treatment of uninfected mice with LPS (NBH + LPS) also induced the expression of pan- and A1-reactive astrocyte-associated transcripts with limited induction of A2- reactive astrocyte-associated transcripts. However, this polarity was not observed in prion-infected mice after LPS treatment. Instead, LPS treatment was accompanied by the elevated expression of pan-, A1- and A2-reactive astrocyte-associated transcripts (prions+LPS; Figure 7). This analysis illustrates how peripheral exposure to pro-inflammatory stimuli such as bacterial LPS could have a significant impact on the phenotype of the reactive astrocytes in the brains of individuals infected with prions. Furthermore, the neuronal damage caused by CNS prion disease may render the infected neuron more susceptible to LPS-induced astrocyte-mediated cytotoxicity [212]

The precise consequences that these LPS-induced effects on the reactive astrocyte transcriptome have on CNS prion disease are uncertain and require further study. Similarly, experiments are also required to determine whether these effects are a consequence of the indirect effects of LPS-stimulation on the microglia [211], or cell-intrinsic responses in the astrocytes themselves, or a combination of the two. While the expression of specific sets of transcripts can be helpful markers to classify cell phenotype [211], their roles in A1- and A2-reactive astrocyte activation also remains to be determined. Despite these uncertainties, this analysis clearly shows that systemic inflammation such as that induced by exposure to LPS can have a significant impact on the reactive astrocytes during CNS prion disease. Other stimuli including the cytokine GM-CSF can also promote the pathogenic activation of astrocytes [220], and could similarly have the potential to exacerbate CNS prion disease.

### 9.3. Pathogen Co-Infection Can Modify CNS Prion Disease

#### 9.3.1. Virus Co-Infections

Studies have revealed how host cellular responses or the pathology caused by certain viral co-infections can affect CNS prion disease pathogenesis. For example, mice succumbed to infection with the Chandler mouse-passaged scrapie isolate much earlier when given a respiratory adenovirus infection towards the clinical phase [221]. Piry virus infection in mice causes a non-lethal arbovirus encephalitis in regions of the brain including the hippocampus. Co-infection of with Piry virus altered the morphology of the microglia in the brains of mice with CNS prion disease. These cells displayed an enhanced ramified appearance, but this did not affect the neurodegeneration or clinical signs [222]. Peripheral co-infection with an apathogenic murine retrovirus (molecular clone Mov3 of Moloney murine leukaemia virus) was also associated with enhanced microglial activation in the brains of mice infected with RML scrapie prions [223]. Here, the enhanced microglial activation in the co-infected mice was accompanied by increased prion clearance and increased lysosomal-autophagy activation in the microglia. However, the effect of retrovirus co-infection on CNS prion disease was transient, as the disease kinetics returned to levels similar to mice infected with prions alone when the microglial activation had declined [223].

Infection with the Friend retrovirus complex can also enhance reactive astrocyte activation in the brains of mice infected with prions [224]. However, the effect this had on disease pathogenesis is uncertain, because although the clinical presentation was altered in the co-infected mice, survival times were not.

#### 9.3.2. Gastrointestinal Helminth Parasites

**Figure 8:**
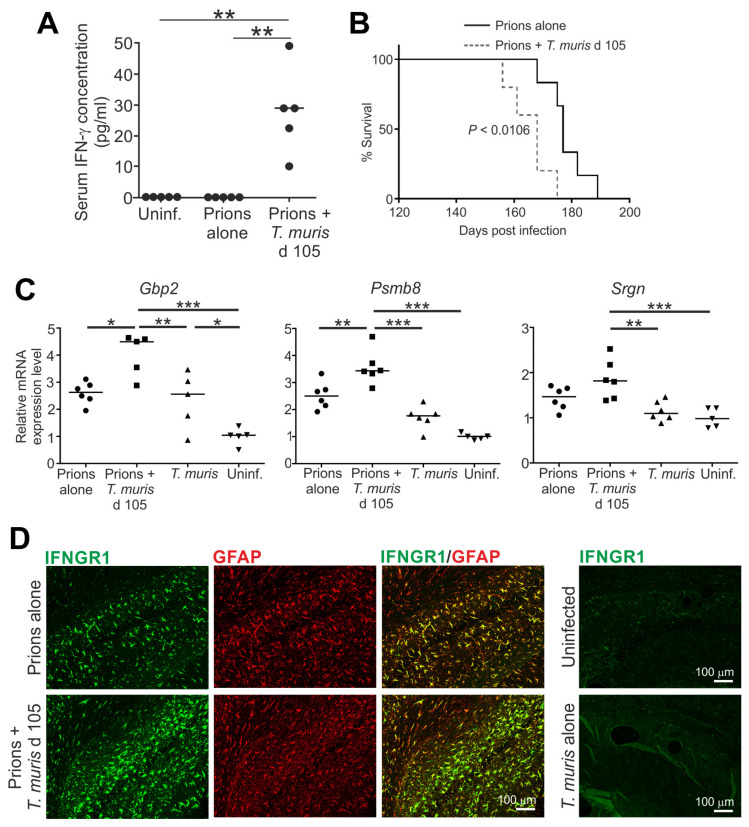
Accelerated CNS prion disease in mice co-infected with a gastrointestinal helminth during the preclinical phase [213]. Mice were injected IC with ME7 scrapie prions and 105 days later they were orally co-infected with ~20 infective *T. muris* eggs. Mice were sampled 35 days later (d 140) or maintained until the development of clinical disease. (**A**) High levels of IFN-γ in the serum of mice co-infected with prions and *T. muris*. (**B**) Survival plot shows accelerated onset of clinical prion disease in mice co-infected with prions and *T. muris*. (**C**) Significantly increased expression of the A1 reactive astrocyte-associated genes *Gbp2*, *Psmb8* and *Srgn* in the brains of mice co-infected prions with *T. muris*. (**D**) Immunofluorescent microscopical detection of high levels of IFNGR1 (green) in GFAP+ reactive astrocytes (red) in the brains of mice infected with prions alone or co-infected with prions and *T. muris*. *, *p* < 0.05; **, *p* < 0.01; ***, *p* < 0.001. Figure adapted from [213] under the terms of the Creative Commons Attribution Licence (CC-BY-4.0; https://creativecommons.org/licenses/by/4.0/).

Little is known of underlying molecular mechanisms responsible for the effects that the virus co-infections described above have on prion disease pathogenesis in the brain. However, a recent study gives insight into how the production of pro-inflammatory cytokines in response to co-infection with a gastrointestinal helminth parasite could accelerate CNS prion disease [213]. The natural mouse gastrointestinal helminth pathogen *T. muris* establishes infection exclusively in the large intestine. When C57Bl/6J mice are infected with a high dose of infective *T. muris* eggs (ca. 200) this induces a protective CD4+ T helper cell type 2 (Th2)-polarized immune response. Conversely, when the mice are infected with a low dose of ca. 20 infected eggs this stimulates a non-protective parasite-specific Th1-polarized response that includes the production of the pro-inflammatory cytokine interferon (IFN)-γ [213,225,226] (Figure 8A). Co-infection of C57Bl/6J mice with a low dose of *T. muris* accelerated clinical prion disease (Figure 8B). The effects of *T. muris* co-infection on CNS prion disease were specific to an IFN-γ-mediated systemic response, as co-infection with a high parasite dose that induced a Th2 response had no effect [213]. The accelerated prion disease in the *T. muris* co-infected mice coincided with the enhanced expression of certain A1 astrocyte-associated genes [213] (Figure 8C). Stimulation of astrocytes with IFN-γ can induce neurotoxic activity [227]. Since the reactive astrocytes in the co-infected mice specifically expressed high levels of IFN-γ receptor 1 (IFNGR1), this suggested that the IFN-γ produced in response to the parasite infection in the intestine had enhanced the neurotoxic phenotype of the reactive astrocytes (Figure 8D). Elevated IFNGR1 expression was also detected in reactive astrocytes in mice infected with prions alone [213]. However, since IFN-γ is not induced in mice infected with prions alone in the steady state [213,228], the induction of IFNGR1 in the reactive astrocytes in response to CNS prion disease most likely primes them to respond to subsequent IFN-γ-mediated stimulation. Furthermore, the A1 astrocyte-associated genes *Gbp2* and *Psmb8* are each inducible in astrocytes by IFN-γ stimulation [229,230].

Although low numbers of CD4+ and CD8+ T cells can infiltrate the brain during CNS prion disease [53], these cells are unlikely to contribute to the neurodegeneration in the steady state [28,30,231]. Interestingly, co-infection with *T. muris* also appeared to enhance the abundance of CD8+ T cells in the brains of mice infected with prions. It is plausible that IFN-γ-mediated stimulation in the co-infected mice enhanced the permeability of the blood–brain barrier enabling these cells to access the CNS [232]. CD8+ T cells with a pro-inflammatory and cytotoxic transcriptome have also been detected in the cerebrospinal fluid and brains of Alzheimer’s disease patients [233]. Other disturbances to the gastrointestinal tract could also affect the abundance of potentially pathogenic T cells in the intestine and have pathological consequences in the CNS. For example, factors produced by certain bacteria strains in the small intestine can induce autoreactive T cells and this is associated with enhanced EAE severity [194]. Further studies are required to explore whether the CD8+ T cells that infiltrate the brains of mice co-infected with prions and *T. muris* also have pathogenic properties.

Infections with gastrointestinal helminth pathogens are common in natural prion disease-susceptible host species. For example, the nematode parasite *Teladorsagia circumcincta* causes infection in the abomasum of sheep. When lambs with natural sheep scrapie were co-infected with *T. circumcincta* during the preclinical phase, this also coincided with shortened prion disease survival times [135]. The effects of the helminth co-infection on the rate of development of the neuropathology were not determined in this study. However, this study raises the possibility that the systemic release of inflammatory mediators in response to helminth infection in the abomasum may have similarly accelerated the development of neuropathology within the CNS.

### 9.4. The Contrasting Effects of Type I Interferons

The production of type I IFN (IFN-α and IFN-β) provides an important first line of defence in host cells against viral infections. However, their production in the brain can have diverging consequences and has been linked to both protective, anti-inflammatory and detrimental effects (reviewed in [234]). Similarly contrasting roles for type I interferons during CNS prion disease have also been reported. Data from one laboratory suggested that type I IFN-mediated signalling via the IFN-α receptor 1 (IFNAR1) may have pathological consequences during CNS prion disease in the steady state, as survival times were extended in *Ifnar1*^−/−^ mice infected with ME7 scrapie prions [235]. Here, the absence of type I IFN signalling during CNS prion disease coincided with suppressed microglial and astrocytic activation, as well as reduced phagocytic activity in the microglia in areas of the brain associated with neurodegeneration. The endoplasmic adaptor molecule, stimulator of IFN genes (STING), is a cytosolic sensor of DNA damage and endoplasmic reticulum stress and a potent inducer of type I IFN [236]. In the absence of STING the production of IFN-β by microglia from the brains of prion-infected mice was blocked. This implies that the STING-mediated detection of damaged neurons by microglia stimulates the production of type I IFN production during CNS prion disease, and this may enhance the rate of neurodegeneration [235]. However, it is important to note that induction of IFN-β has not been observed in patients with sCJD or in independent studies of mice infected with ME7 scrapie prions [237,238].

Conversely, data from another series of studies have proposed that type I IFN may play a protective role [239,240]. In contrast to data above [235], prion disease developed earlier in *Ifnar1*^−/−^ mice infected with 22L scrapie prions, whereas treatment with a type I IFN antagonist (RO8191) extended survival times [240]. The reasons behind these discrepancies are not immediately apparent, but further studies may help to identify specific type I IFN regulators that influence the rate of development of CNS prion disease in certain individuals.

### 9.5. COVID-19

Infection with the severe acute respiratory syndrome coronavirus 2 (SARS-CoV-2) causes coronavirus disease 2019 (COVID-19) [241]. This highly infectious disease has rapidly spread worldwide and, at the time of writing (August 2020) had been attributed to at least 240,000 deaths [242]. A striking characteristic of this infection in the UK was that >90% of COVID-19-related deaths in the UK occurred in individuals who were >60 years old [243]. In these patients, dementia was identified as a highly significant co-morbidity factor for risk of hospitalized COVID-19 and death [244,245]. Whether COVID-19 enhances the progression of the neurodegeneration in these elderly individuals remains to be determined. However, a range of studies have discussed how systemic infection and gastrointestinal infections could exacerbate the cognitive decline or neuropathology in patients with Alzheimer’s disease [246,247], Parkinson’s disease [248] and amyotrophic lateral sclerosis or frontotemporal dementia [249].

A recent case report of a > 60-year-old patient reported that the onset of clinical sCJD signs occurred concurrently with those of COVID-19 [250]. Further studies are clearly necessary to investigate this association, and coincidence cannot be entirely excluded. However, considering data discussed in the above sections, it is tempting to speculate that the strong inflammatory responses that have been described in patients with severe COVID-19 disease [251] could exacerbate the neuropathology. For example, viral load in COVID-19 patients correlates highly with serum TNF-α levels, and severe COVID-19 disease is associated with high serum TNF-α [251]. As described above (Section 9.2.1), TNF-α is an important inducer of neurotoxic A1 reactive astrocytes in the brain [211]. Furthermore, TNF-α administration exacerbates the clinical signs in mice infected with prions [252] and exaggerates the reactive astrocyte activation [219]. Viral load in COVID-19 patients also correlates highly with serum IFN-γ levels [251], and those requiring intensive care treatment have high serum IFN-γ levels [253]. This cytokine can similarly stimulate neurotoxic reactive astrocyte activation [227], and IFNGR1 expression is upregulated in reactive astrocytes during CNS prion disease [213], in aging human brains and patients with Parkinson’s disease and Alzheimer’s disease [227,254]. Thus, it is plausible that the systemic production of high levels of pro-inflammatory mediators such as TNF-α and IFN-γ in response to infection with the SARS-Cov-2 virus could accelerate the neurodegeneration in patients with concurrent prion disease or other neurodegenerative disorders.

## 10. Conclusions

Despite decades of intensive research, there are still no effective treatments that can be used to block prion disease susceptibility or intervene in the disease process. As a consequence, the onset of clinical prion disease is invariably fatal in affected individuals. Many of the studies discussed throughout this review show how modulation of immune cell abundance or function can have protective effects on prion disease pathogenesis and susceptibility. Similar changes, either around the time of prion exposure or towards the clinical phase, could influence an individual’s risk of being infected and developing disease. This could explain why some individuals develop clinical disease and others do not, despite exposure to similar amounts of prions. For example, the UK population is likely to have been widely exposed to the BSE agent through the consumption of contaminated food during the BSE epidemic. Despite this, the incidence of probable and definite clinical vCJD cases has fortunately been relatively low. Since data from BSE transmission to transgenic mice expressing human PrP^C^ indicate that there is a significant barrier to the cross-species transmission of BSE prions to humans (known as the species barrier effect) [255], it is feasible that, in some individuals, the effects of inflammation around the time of exposure may have increased their susceptibility to oral prion infection.

Conversely, a range of studies have raised the credible prospect that immunotherapeutic approaches, such as passive or active immunisation, may have efficacy against these currently untreatable and devastating disorders (reviewed in [256,257]). For example, transgenic mice that are engineered to secrete anti-PrP antibodies are protected against peripheral prion infections [258]. Passive immunisation with monoclonal anti-PrP antibodies is similarly protective in mice but large quantities of anti-PrP antibodies were administered for the duration of the experiment [259]. At the time of writing, a clinical trial in the UK was underway to test the efficacy of anti-PrP antibody treatment (using monoclonal antibody PRN100) in six sCJD patients [260]. Of course, the blood–brain barrier limits the entry of large blood-borne molecules such as antibodies into the brain [261]. Therefore, to be effective against the CNS phase of prion disease, these must be administered directly into the CNS of affected individuals, although use of camelid-derived PrP-specific heavy chain antibodies, or nanobodies, may offer an alternative approach [262].

However, since each of these examples involve the administration of antibodies against the PrP molecule, the potential for them to bind to cellular PrP^C^ (especially on neurons) and cause autoimmunity or neurotoxicity must be considered [263,264]. Fortunately a detailed assessment of the impact of the binding of anti-PrP antibodies to different domains of the PrP^C^ molecule offers useful advice for future antibody-based treatments: antibodies that bind to the globular protein domain can be neurotoxic, whereas those that bind to the flexible tail of PrP^C^ appear to be neuroprotective [263]. Antibodies are considered to provide protection through mechanisms including the elimination of prions from affected tissue or blocking PrP^C^–PrP^Sc^ conversion. However, stimulation with certain anti-PrP antibodies can induce anti-inflammatory macrophage polarisation and, by doing so, protect mice from an otherwise lethal influenza infection [10]. It will be interesting to determine whether similar anti-PrP antibody-mediated effects could indirectly limit the neurotoxic activation of microglia during prion disease [147,168].

Prion disease has been transmitted in natural host species, including humans, via the oral route after ingestion of food or pasture contaminated with prions (discussed in [16]). An effective anti-prion vaccine will therefore need to induce both mucosal (IgA) response and systemic (IgG) antibody responses to protect against prion invasion from the intestine and the subsequent effects of prion accumulation in tissues. Fortunately, experimental studies in mice and white-tailed deer have provided proof of principle that mucosal immunisation against PrP^C^ can provide partial protection against orally-acquired prion disease [265,266]. The enterocytes that line the intestine can transcytose IgG into the intestinal lumen, and can subsequently transport IgG-antigen complexes back across the epithelium by a process known as reverse transcytosis [267]. It remains to be determined whether anti-PrP^C^ antibodies might in some instances enhance the transcytosis of prions across into Peyer’s patches and by doing so increase disease susceptibility [268].

**Figure 9:**
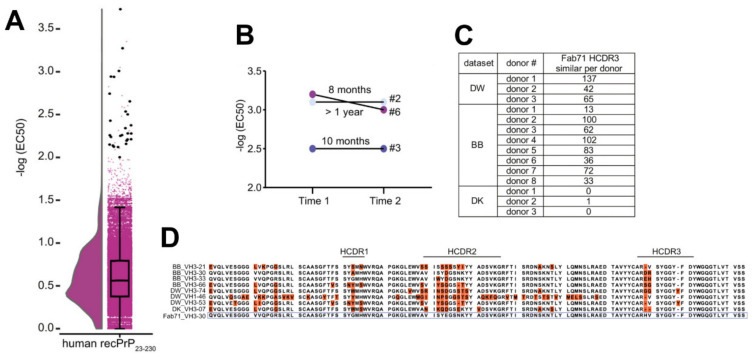
Detection of protective anti-PrP antibodies in human serum and immunoglobulin repertoires [270]. (**A**) Violin plot/box plot showing detection of 21 individuals (solid black circles) with high-titre anti-PrP antibody titres in serum samples from 41,718 Swiss hospital patients. (**B**) Anti-PrP reactivity was maintained for at least 8 months in some individuals. (**C**) Identification of HCDR3 sequences similar to a synthetic phage-derived anti-PrP Fab clone (Fab71) in three different datasets of human antibody repertoires from healthy donors. (**D**) The amino acid sequence of Fab17 VH3-30 compared to the HCDR3 regions identified in the different healthy donor databases that differed from Fab71 by ≤3 residues. Orange boxes indicate amino acids distinct from Fab71. Adapted from [270] under the terms of the Creative Commons Attribution Licence (CC-BY-4.0; https://creativecommons.org/licenses/by/4.0/).

Finally, natural, rare instances of high-titre, anti-PrP^C^ antibodies have been detected in the bloodstream of some humans. In one study, serum samples were analysed from 128 individuals with various pathogenic prion disease-associated *PRNP* mutation and 78 control individuals that lacked these mutations but had a positive family history of genetic prion disease [269]. While disease-associated *PRNP* mutations did not generally stimulate antibody responses to PrP^C^, some individuals in this study did have anti-PrP^C^ antibodies (IgG subclass) in their sera. However, the presence and titres of these anti-PrP^C^ antibodies were similar across the subject groups, arguing against them providing protection against prion disease or directly contributing to the neurodegeneration. In a separate study, serum samples from over 48,000 Swiss hospital patients were screened. Amongst this large collection of samples, 21 individuals were identified with potentially-protective high-titre anti-PrP antibody titres [270] (Figure 9A). In some individuals, these titres were maintained for at least 8 months (Figure 9B). An additional genetic screen of large-scale antibody repertoire sequences derived from circulating and naïve B cells from healthy donors also identified the existence of some potentially-protective PrP^C^-specific antibody clones [270] (Figure 9C). Further research is now necessary to determine whether these are sufficient to offer in protection to these individuals against prion infections in vivo. The demonstration that potentially-protective anti-PrP antibodies were detected in some individuals without obvious signs of neurotoxicity raises hope that antibody-based intervention strategies against prion diseases may be tolerated without significant adverse reactions.

## Abbreviations

BSE	Bovine spongiform encephalopathy
cDC	Conventional dendritic cell
CNS	Central nervous system
CSF1/R	Colony-stimulating factor 1/receptor
FAE	Follicle-associated epithelium
FDC	Follicular dendritic cell
GALT	Gut-associated lymphoid tissue
GSS	Gerstmann–Sträussler–Scheinker disease
IC	Intracerebral
IFN	Interferon
IFNGR1	Interferon gamma receptor 1
IL	Interleukin
IP	Intraperitoneal
LPS	Lipopolysaccharide
MFGE8	Milk fat globule epidermal growth factor 8
MHC	Major histocompatibility complex
MNP	Mononuclear phagocyte
PrP^C^	Cellular PrP isoform
PrP^Sc^	Prion disease-specific PrP isoform
sCJD	Sporadic Creutzfeldt–Jakob disease
SED	Subepithelial dome
SFB	Segmented filamentous bacteria
SLO	Secondary lymphoid organ
TGF	Transforming growth factor
TNF	Tumour necrosis factor
UPR	Unfolded protein response
vCJD	Variant Creutzfeldt–Jakob disease
